# Fiber Optic Sensors for Harsh and High Radiation Environments in Aerospace Applications

**DOI:** 10.3390/s23052512

**Published:** 2023-02-24

**Authors:** Alberto Rovera, Alexandru Tancau, Nadia Boetti, Matteo D. L. Dalla Vedova, Paolo Maggiore, Davide Janner

**Affiliations:** 1 Politecnico di Torino, C.so Duca degli Abruzzi 24, 10129 Torino, Italy; 2 LINKS Foundation, Via Pier Carlo Boggio 61, 10138 Torino, Italy

**Keywords:** fiber optics sensor, radiation, harsh environment

## Abstract

In the upcoming space revolutions aiming at the implementation of automated, smart, and self-aware crewless vehicles and reusable spacecraft, sensors play a significant role in the control systems. In particular, fiber optic sensors, with their small footprint and electromagnetic immunity, represent a great opportunity in aerospace. The radiation environment and the harsh conditions in which these sensors will operate represent a challenge for the potential user in the aerospace vehicle design and the fiber optic sensor specialist. We present a review that aims to be a primer in the field of fiber optic sensors in radiation environments for aerospace. We review the main aerospace requirements and their relationship with fiber optics. We also present a brief overview of fiber optics and sensors based on them. Finally, we present different examples of applications in radiation environments for aerospace applications.

## 1. Introduction

**Figure 1:**
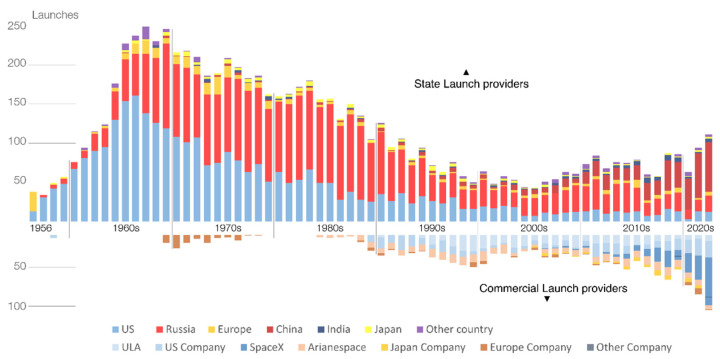
Orbital launches from 1956 to 2022 from the main operators. Inspired by [6].

In the next decades, space will be one of the new frontiers of development for the private and public sectors. New projects, such as Project Artemis [1] with the Deep Space Gateway [2] and a moon base [3], will revolutionize the space economy by leveraging on a more sustained involvement of private companies that now have cheaper access to space after the mission Inspiration 4 [4]. Indeed, space is already considered an extension of the current domestic and business environment both in geopolitical and economic terms [5]. This trend is well displayed by the historical evolution of the number of satellite launches in Figure 1 and elaborated with the methodology in [6], where we report the number of orbital launches since the 1950s for different operators.

A fundamental aspect of this upcoming space revolution will focus on implementing automated, smart, and self-aware crewless vehicles and reusable spacecraft. This point must leverage the key enabling technology of advanced and radiation-compliant sensors both for spacecraft automation and for the astronauts’ health and security. For instance, a future Mars colony should survive in a harsh environment where radiation can reach very high levels with solar flares. In such a context, a system that can sense any strain, temperature, and radiation while being able to use these data to foresee possible failures is a key enabler for a successful space quest and colonization.

Space is a harsh environment with extreme temperatures, high levels of radiation, and an ultra-high vacuum. Each of these characteristics represents a threat to the spacecraft and the astronauts. Electronic systems are highly susceptible to electromagnetic fields and radiation. This is a serious concern for components due to their ever-increasing miniaturization, which boosts their sensitivity to those conditions. Optical fibers can overcome such issues in the space environment. Indeed, their successful usage in space for data transfer [7] showed their usability, increasing the interest in new ways to utilize them as optical sensors. This fact drives the increasing interest of researchers and industries of these technologies, while their diffusion in other fields of application, aside from space, leads to improved maturity and lower costs.

The increased interest in optical fiber sensors (OFS) is demonstrated by the fact that market forecasts of the combined point and distributed sensors exceeded their expected growth. In 2019, the market’s projection was forecast to be over 1.3 billion by 2023 [8], but in 2021, the combined market size reached 2.358 billion [9,10]. The main contribution to such an unanticipated increment comes from the increase of the market share of other fields of application, different from the traditional oil and gas. Indeed, in 2014, 49% of the market was associated with oil and gas with its significant fluctuations, but by 2023, it will decrease to a 19% share. The fields widening their market share are energy, infrastructure, and aerospace.

Given this dynamic growth of the space field for OFS, and the peculiar characteristics of its harsh environment that make OFS deployment challenging, an updated review can give an overview of the state of the art in this field and their latest applications. In this context, the present review aims to be a primer to the topic, addressed towards the non-experts coming from the fields of mechanical/aerospace engineering or the fiber optics world looking for applications in radiation environments for fiber sensors. 

At first, we review the space environment and its constraints, particularly the extreme temperatures and radiation that OFS should withstand. Afterward, the different types of fibers and optical fiber sensors are reviewed in Section 1.2 and Section 2, along with their functioning mechanism and production technologies. The radiation response and the radiation hardening techniques are explained in Section 3 and Section 4, respectively, focusing on the most critical aspects to increase their security and applicability for long-term space missions. Finally, in Section 5, we present some application cases and a few considerations for the future outlook of OFS in space, radiation, and harsh environments.

### 1.1. Space Environment

In the space environment, continuous material degradation, contamination, thermal changes, radiation damage excitation, spacecraft glow, charging, and induced background interference are the most critical hazards that make it a harsh environment. These effects originate from many sources and physical phenomena such as orbital debris, meteoroids, mechanical vibrations, significant thermal variations, protons, electrons, neutrons, high-energy particles, X-rays, and γ-rays. Therefore, in each space mission, devices and systems need to be tailored for these hazards, starting from the material choice to the design and up to the final assembly. This process is carried out considering the mission’s length and the lifetime required. The latest trends in this respect are pushing the performance requirements for aerospace to shift towards lighter exotic materials and more complex spacecraft systems. 

Since the late 1970s, optical fiber sensors have attracted attention as one of the most promising technologies for aerospace applications due to their peculiar properties, such as small size, lightweight, immunity to electromagnetic fields, multiplexing capability, and fast response (see Section 4 and Section 5). From the early days of their usage, the effects of harsh environments and, specifically, radiation on optical fibers have been studied. Yet, the large number of glass compositions and configurations made it impossible to completely investigate radiation effects on all the fibers, delaying the acceptance of such potentially disruptive technology for serious reliability concerns.

Similar to all technology that needs to be validated for space and aims to be adopted as a standard, fiber optics also underwent many assessments carried out by several organizations [11]. The standardized protocol must follow these steps:

1. Define the environment of the mission;
2. Evaluate the interaction between environment and space assets;
3. Define the requirements and their criticalities;
4. Assess the design and the performance characteristics of components;
5. Improve the design and performance along with the definition of the design margins and the risks assessments;
6. Iterate the process with updated knowledge.

Since the starting point is the definition of the mission’s environment, we recall the main ones briefly: Low Earth Orbit (LEO), Middle Earth Orbit (MEO), Geosynchronous (GEO), Geosynchronous Transfer Orbit (GTO), interplanetary and other planets. 

The different orbits are characterized by their distance from the earth. LEO orbit, the one where the international space station and most earth observation satellites are located, is between 200 and 2000 km from the earth’s surface. MEO orbits are between 2000 and 35,586 km, and they are used by navigation satellites such as the European Galileo system. GEO orbit satellites are at an altitude of 35,786 km, while GTO orbit satellites have a strong elliptical orbit between 42,164 and 35,786 km. These orbits are typically used by telecommunication satellites [12,13,14].

Each of those environments presents a different environment with significant variations; however, all space missions share a common characteristic: long durations. Usually, their lifetime ranges from 7 to 15 years for GEO missions, but it can be even longer [15]. With this primary goal of mission duration, a differentiation is operated in terms of requirements according to the operative environment of the missions, with particular attention paid to the sources of degradation or hazards. As summarized in Table 1, multiple hazards are present in the space environment as a potential source of damage to the structure and fiber optics. Among all the hazards, ionizing radiation and particles are the most critical for OFS. Indeed, three primary sources of particles and radiation interact with optical fibers and are as follows: trapped particles, cosmic rays, and solar energetic particles. Fiber optics degrade mainly through the ionizing process, making solar particles and trapped particles the most dangerous. 

**Figure 2:**
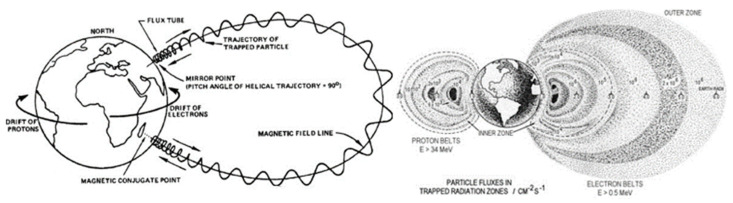
Gyration, bounce, and drift movement of trapped particles (**left**) [20] and flux densities of trapped electrons and protons (**right**) [21].

Fortunately, trapped particles are only present in a small zone around planets. Indeed, around the earth and any planet with a magnetosphere, there is a toroidal region called trapped radiation belts or Van Hallen radiation belts. Here, charged particles are produced or arrive, and they become trapped, gyrating and traveling along the field lines of the magnetic field. The Earth’s radiation belts range between 200 km and 75,000 km from the surface, and the trapped particles have three components: gyration, bounce, and drift. This causes trapped electrons and protons to have different energies and different spatial distributions, as shown in Figure 2. Electrons reaching up to 7 MeV and protons up to 600 MeV constitute a significant hazard for spacecraft. There are different models for the distribution of the particles and for engineering applications; two static models are mainly used by NASA, AP-8 and AE-8 [18], and improved models (AE-9, AP-9) have been created [17]. Due to a large number of models available, ESA tried to specify the required and recommended method, model, and data for space engineering to make it consistent and improve any space assets’ survivability and performance. All these standards are illustrated in the report ESCC-E-ST-10-04C Rev.1 [19]. 

Solar particles (or SPE, solar particle event) are also present outside radiation belts, which induce damage by ionizing the optical fibers. They are generated by solar flares and coronal mass ejections, which produce protons, electrons, neutrons, X-rays, γ-rays, and heavy ions. These events potentially lead to high fluxes and fluences of particles with energies variable but inferior to galactic cosmic rays. Even if those energies are inferior to cosmic rays, their fluxes are higher, and SPE has the most impact on fiber-based devices. Galactic cosmic rays (or GCR) are a continuous flow of charged particles that arrives from outside the solar system, and they are primarily atomic nuclei stripped of their electrons [22]. GCRs have more importance for single event effects for higher atomic number elements due to their high energy. This poses a great risk for microelectronics but is of less concern for optical fibers, where the cumulative effect of radiation (called total ionizing dose, TID) has a more significant impact. For these reasons, in the literature, much attention is dedicated to the effect of ionizing radiation on fibers and how to make them resistant to damage resulting in the malfunction of OFSs. This process is called radiation hardening, and in the following, we detail the main issues that OFSs face in the aerospace environment.

### 1.2. Types of Optical Fibers

**Figure 3:**
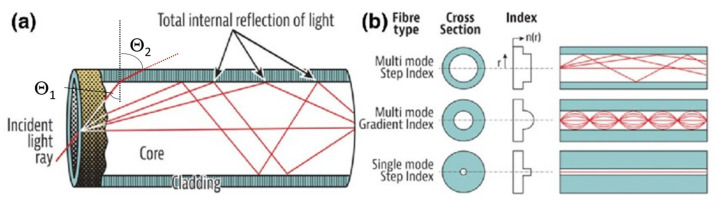
(**a**) The TIR mechanism and (**b**) the typical structure of multi-mode (MM) fibers with a larger core able to propagate multiple modes, and single-mode (SM), which are the most common with a small core [23].

Many different types of optical fibers are available on the market today with many different geometries or materials. Silica-based (SiO2) optical fibers that guide light by a total internal reflection mechanism (TIR) are the most commonly used. This mechanism is shown in Figure 3.

This propagation occurs through multiple reflections at the interface between the core and cladding that is quantified by the so-called Snell’s law:

n_CORE_ sin(θ_1_) = n_CLADDING_ sin(θfor tens of km with a typical resolution of_2_), (1)

where:

- n_CORE_ is the refractive index of the material composing the core;
- n_CLADDING_ is the refractive index of the material composing the cladding;
- ϴ_1_ is the angle of incidence of the light ray at the core-cladding interface;
- ϴ_2_ is the angle of refraction of the light ray after reaching the interface.

Snell’s law describes and defines the physical phenomenon that, when a ray of light changes the material within which it propagates, it also changes its direction of propagation from being ϴ_1_ to ϴ_2_. To achieve total internal reflection, the condition is that n_CORE_ must be larger than n_CLADDING_ since, in that case, Θ_2_ is larger than 90°, implying that the refracted ray bounces back inside the core, thus allowing the transmission of information. Under this condition, we can also derive the maximum angle (called critical angle) at which light can enter the fiber and be confined in the core. Light coming at smaller angles is confined in the core and thus guided.

The refractive index (RI) difference between the core and the cladding is obtained by doping the core glass with chemical elements such as germanium or phosphorus, even according to a geometrical profile that defines the allowed guided modes (see Figure 3b). Other materials besides silica can be used as a matrix if other characteristics such as transparency window, the high solubility of rare earth ions, or higher refractive index are desired, such as tellurite, fluoride, and phosphate [24]. According to the materials present in the core, different and complex radiation responses can be obtained by determining the fiber response to radiation.

To provide mechanical and chemical protection to the fiber (core/cladding), the cladding is typically surrounded by a coating, normally made of a polymer such as a polyimide or a polyacrylate. Different protective coatings can be added on top of the polymer coating or in substitution for applications in harsh environments. For example, a metallic layer of aluminum or gold deposited on a silica fiber can increase temperature or radiation resistance [25].

Several classes of optical fibers are obtained by changing the shape and RI profile of the core or the cladding. The primary differentiation in the actual market is between standard and specialty optical fibers [26]. Specialty optical fibers, with different materials or more complex designs, are very interesting for sensors, amplifiers, or fiber-based lasers and have shown future growth potential and growing interest by industries in the last few years. However, their market share is reduced to a few percent compared to the over 50% taken by standard or conventional fiber optics.

Conventional fibers are the most common and can be employed in data transfer, diagnostics, and sensing applications. They are widely used in telecommunications where long lengths are required, and the optical losses need to be as low as possible. Indeed, 0.14 dB/km (1550 nm) losses were achieved recently [27]. Among them, single mode (SM) fibers have a core diameter between 8 and 10 μm, with a cladding diameter of 125 μm. They are commonly made with a step index RI profile (see Figure 3b), where an abrupt difference in refractive index causes the total internal reflection. Doping elements such as Ge, F, Al, etc., can modify the refractive index of the clad and core, and the exact RI is obtained by dosing these dopants in the fabrication process. Most SM applications are meant for long-distance communication and sensors due to their low dispersion and good optical signal integrity. Their response to radiation has been extensively studied over the years in relation to their fabrication process and doping elements. For instance, fluorine-doped fibers showed outstanding resistance to radiation environments by having a radiation-induced loss lower than 5 dB/km [28]. Although such a value can be considered a high loss for an SM fiber in telecommunication applications, the short lengths typically needed for space applications make it a good result. Other researchers found that the fabrication parameters have a significant correlation to the radiation resistance of SM fibers; however, more experiments are needed to provide a sound prediction for the radiation-induced attenuation based on these parameters.

Multimode (MM) fibers have larger diameter cores than SM, more than 50 μm, while the clad might have diameters ranging from 125 μm to 400 μm. They are used for short distances compared to SM fibers in the telecommunication field and can propagate multiple modes. Thanks to the greater numerical aperture compared to SM, it is easier to couple light through them, and cheaper light sources such as LEDs can be connected to such fibers more efficiently [29]. MM fibers are less common for scientific research and only few studies were performed on their radiation behavior [30,31].

**Figure 4:**
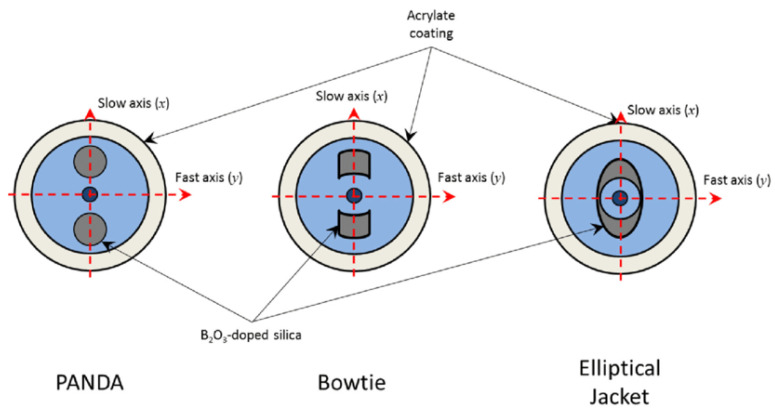
Examples of the most common types of PM fibers [34].

Polarization-maintaining fibers (PMFs) are SM fibers in which the guided light maintains its linear polarization state. This is important for some applications such as interferometric sensors, gyroscopes, and coherent communications [32]. There are different ways to obtain polarization maintenance in PMF, as reported in Figure 4. The most common approaches are noncircular core and asymmetrical lateral stress. The latter is used in PANDA fibers in which the asymmetrical lateral stress is introduced by two stress rods made of silica glass modified with boron and placed inside the clad [33]. The presence of stress along one direction breaks the symmetry of the core, introducing a birefringence where the RI has a slightly higher value (slow axis) along one direction. This allows for decoupling the modes in the orthogonal axes and maintaining the polarization if the light is linearly polarized along the direction of one of these two main axes.

PM fibers are especially interesting for aerospace applications since they are a key component of fiber optic gyroscopes (FOGs). Recent improvement in radiation hardening by composition has been made. It has been observed that N-doped fibers under an irradiation of 2–10 kGy (higher than normal space conditions) have losses that do not exceed 5–10 dB/km, an order of magnitude better than standard SM Ge-doped silica core fibers. This makes them an interesting alternative for aerospace applications in harsh environments [35]. The disadvantage of these fibers is their high cost derived from their complex structure and fabrication.

**Figure 5:**
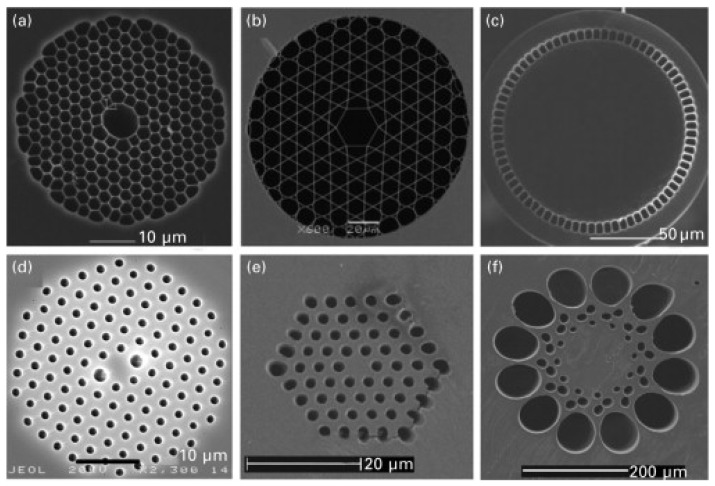
Examples of microstructured optical fibers (MOFs): (**a**) silica hollow-core photonic bandgap fiber (Humbert et al., 2004); (**b**) silica hollow-core Kagome lattice fibre (Couny et al., 2006) which is guided by the inhibited coupling mechanism (Argyros and Pla, 2007); (**c**) silica hi-NA fiber (Wadsworth et al., 2004b), © IEEE; (**d**) silica birefringent fiber (Xiong and Wadsworth, 2008); (**e**) single-mode mPOF; (**f**) high-bandwidth mPOF [39].

First proposed in the early 1970s by Kaiser et al. [36] to reduce losses, microstructured optical fibers (MOFs), such as the ones in Figure 5, were first only produced in the 2000s due to their complexity of production. There are two main categories of MOFs: structured optical fibers based on TIR effect to guide light and photonic crystal (or bandgap) fibers, which are treated separately as they leverage a different light-guiding mechanism.

Microstructured fibers have an outer cladding with a core made of glass and air with specific geometries. The TIR mechanism is achieved by the difference in refractive index between the core and air. The advantage they have is that they are made of a single material, e.g., pure silica, for the entire fiber [37]. As reported by Girard et al. [38], MOFs showed comparable losses to pure silica fibers in a radiation environment such as the one produced by fusion by inertial confinement (where a microcapsule, filled with deuterium and tritium, is heated and compressed until nuclear fusion ignites). Therefore, they are good candidates to be used as sensors for environments with high doses of radiation. Still, there are many unknown mechanisms in their radiation-induced attenuation (RIA), and additional research needs to be conducted to improve their radiation hardening.

Photonic bandgap fibers (PBGF) are a subcategory of MOFs that use air as a medium to guide the light and a micro-structured web-like cladding of glass (Figure 5a,b). They are based on photonic crystals. A two-dimensional periodic optical nanostructure creates a photonic bandgap, where light propagation is prevented from occurring outside the hollow core and light is kept inside. Being more recent, with a high cost of production and a difficult fabrication process, they are still not widespread even if they show good results under irradiation, especially under steady state γ-rays [40,41].

Rare-earth doped fibers are important in space applications since they are key for IR-fiber lasers, amplifiers, and FOGs. They are active fibers where the doping elements are not used to modify the refractive index but to absorb a pump light of a certain wavelength, typically shorter, allowing the stimulated emission of light in lasers and optical amplifiers. The rare earth doping elements present in the core tend to have strong interactions with radiation, generating complex mechanisms of degradation. Many studies have been carried out on the radiation response of this category of fibers in the last two decades [42,43,44].

## 2. Optical Fiber Sensor Types

Each optical fiber sensor (OFS) class targets different applications based on the sensing mechanism. The main categories of OFS can be identified as point sensors and distributed sensors. In point sensors, a specific and spatially limited part of the fiber, e.g., a Bragg grating, is sensitive to external stimuli, while in distributed ones, all the fiber can act as a detector. This section compares the main categories and classes of OFS, their sensing mechanism, and their advantages with particular attention applications in the aerospace environment. A detailed description of all the sensing mechanisms is outside this review’s scope but can be found in the references herein. 

### 2.1. Point Sensors

Among the point sensors, the most widely used are fiber Bragg gratings (FBG) and long period gratings (LPG). Even though each type of these OFS has its niche of applications, FBGs are the most studied optical fiber sensors under irradiation and have recently acquired a large market share. The growth forecast of the FBG market in 2028 is expected to reach a global market size of USD 5167.4 million with a 23.9% compound annual growth rate [9]. 

**Figure 6:**
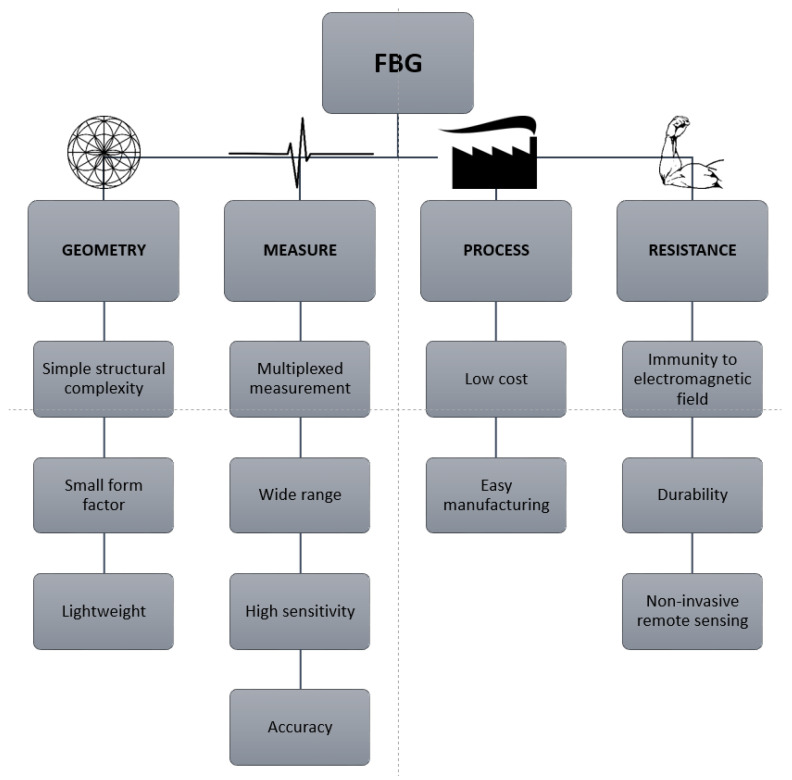
Fiber Bragg grating sensor characteristics [15,45,46].

The main advantages of FBGs are summarized in Figure 6, divided by the different features driving the optimal sensor selection in the various applications. Although some of these advantages also apply to distributed sensors, point sensors generally benefit from a lower cost for the interrogators and a much higher sensitivity. In the following, we review the FBG mechanisms and their relevant fabrication techniques, particularly for degradation mechanisms in the presence of radiation.

#### 2.1.1. Fiber Bragg Gratings

**Figure 7:**
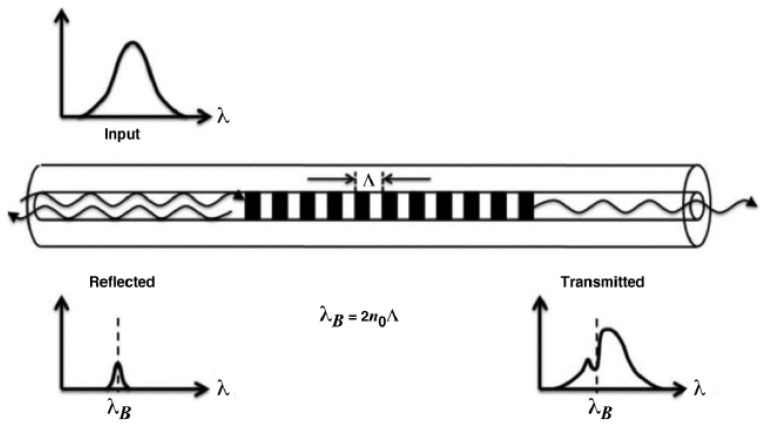
Bragg grating design and mechanism [48].

Fiber Bragg gratings can be seen as reflectors for a very narrowband set of wavelengths (typically 0.2–0.5 nm) and are inscribed directly on the optical fiber’s core. The mechanism of reflection is obtained through a periodic variation (period Λ) of the refractive index of the order Δn = 10^−4^–10^−3^, which creates a resonance for a specific Bragg wavelength, producing the peak in reflection related to the periodicity Λ (see Figure 7). The relationship between the Bragg wavelength λ_B_ and the periodicity of the index variation in fiber Λ is given by:

λ_B_ = 2*n*_eff_Λ, (2)

where *n* is the effective refractive index in the core of the fiber. On average, the effective refractive index is in the range *n* = 1.4–1.5, and Bragg gratings are inscribed (or “written”) on a single mode fiber’s core with a period Λ typically from 0.5 μm to a few μm. This allows for precisely tuning the Bragg wavelength in the spectral region of interest for detection and sensing [47]. 

Since the Bragg wavelength depends on the refractive index (see Equation (2)), its change causes the shift of the reflection peak. The phenomena that produce a change in the fiber’s RI are temperature variation and mechanical strain. Calibrating the response of FBG with respect to these two parameters allows them to be used as sensors for these two physical quantities. The typical strain sensitivity of commercial FBGs is about 1 pm/ με and for temperature is 10 pm/°C. Still, these values can be increased by using special pre- and post-treatments, fiber coating, and packaging.

Since the periodic variation in the core has a typical extension of a few mm along the fiber, FBGs are considered single point sensors, meaning that only the written part acts as a transducer and responds to environmental changes. Moreover, the wavelength selective response of the FBG related to the periodicity Λ allows for chaining multiple FBGs at different Λ along the same fiber. Such multiplexed interrogation systems can be seen as multipoint sensors or a quasi-distributed OFS if they are tightly spaced [49].

To produce FBGs, different manufacturing techniques have been developed and optimized, and the most common today are [15,47,50,51]:

1. Phase mask (PhM);
2. Point by point (PbP);
3. Free space interferometry;
4. Continuous core scanning.

The first FBG was obtained by Hill et al. [50], who found that the core of standard telecommunication fibers was photosensitive due to the presence of germanium inside the core. Indeed, when exposed to high-intensity visible or UV light, its refractive index would increase proportionally to the light intensity. By modulating the light, it was possible to change the refractive index, creating a periodic pattern. 

**Figure 8:**
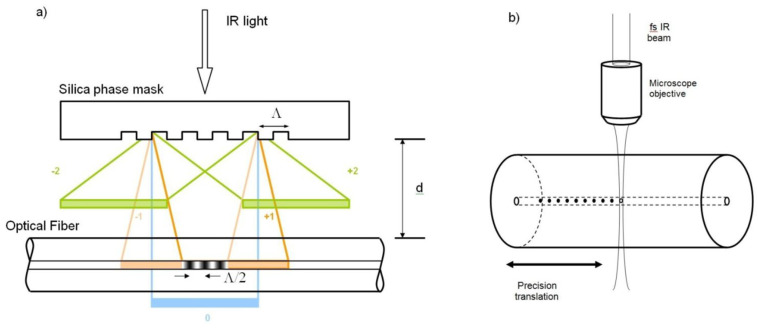
Schematic representation of two common technologies: (**a**) phase mask inscription with an fs-IR laser and (**b**) the PbP technique [52].

In the phase mask technique (Figure 8a), a phase mask is interposed between the laser and the fiber, almost in contact with the fiber itself. The mask determines the grating period, producing a diffraction pattern directly related to the spatial modulation of the refractive index to be produced for the FBG. Such a technique is highly reproducible, but each mask can usually produce only gratings working at a specific wavelength, thus requiring multiple masks to cover a reasonable portion of the spectrum where the FBG should operate. 

The PbP technique generates each grating element controlling the laser parameters and the fiber movement by focusing the laser light on a single point where the index modification is realized. It is based on the nonlinear absorption of an ultrashort laser pulse (Figure 8b). Such absorption produces damage in the material creating zones inside the fiber where the density is slightly changed. By patterning each point, there is fine control of the periodicity of the FBG, leading to a more flexible pattern generation technique. However, such a technique has a much lower throughput with respect to the phase mask one. 

OFSs are commonly used for strain and temperature monitoring efficiently, but they can be functionalized for other applications, as are detailed in Section 4.1. 

#### 2.1.2. Grating Types

Type I gratings are also referred to as standard gratings, and they were the first type of FBG to be produced. They have a positive refractive index change created through the reaction between the UV/IR laser and the color centers in the core glass [52]. The first Bragg produced was Type I-UV and used the oxygen deficiency defect centers in a germanosilicate fiber. To increase the photosensitivity, the core needs to be doped with elements such as Ge [53], and if a higher refractive index change is needed, H_2_ loading can be used. Highly photosensitive Type I hydrogenated fibers are called Type IA. 

All Type I FBGs present a temperature-dependent bond-restructuring phenomenon. Indeed, if the FBG is annealed at temperatures below 450 °C, the thermal treatment stabilizes the grating through the thermal depopulation of trapped excited states. However, for temperatures higher than 450 °C, most of the refractive index changes are destroyed by breakage of the formed color centers, especially Type IA.

When a high peak power short-pulsed laser is used to produce the grating over a certain damage threshold of the fiber, Type II FBGs are created. This type of FBG exhibits higher reflectance (<99%) and larger peak width, thus reducing the typical grating length. With UV-lasers, the damage threshold is surpassed, and physical damage is produced between the core and clad, while for IR-lasers, the gratings are realized by the densification of silica. The sensors based on Type II FBGs are more stable and can be used at temperatures over 1000 °C, but they have a lower quality of the reflection spectra, and the fabrication process tends to reduce the overall mechanical strength.

Type I gratings are the former Type IIA, and they are produced with a UV laser on highly stressed Ge-doped fibers. Under certain conditions, a Type I grating starts to grow and then decreases its strength as the gratings saturate. If the exposure continues, a secondary grating starts growing with a negative RI change [54]. Type I gratings are stable up to 700 °C [55].

Regenerated gratings are obtained from the thermal erasure of the grating. The most common way to produce a regenerated grating is using a Type I grating enhanced through hydrogen loading to increase its photosensitivity. The regeneration process is carried out at room temperature, exposing the FBG to high-pressure hydrogen [56]. After UV inscription creates a Type I, the grating is annealed at a temperature between 600 °C and 700 °C, erasing the Type I FBG, but if the heating continues at an even higher temperature, a new grating appears at a longer wavelength. This grating is stable at high temperatures, even above 1000 °C, but its spectral response weakens by order of magnitude, producing a low-reflectivity FBG. The full physical details of the mechanism underlying the regeneration of the FBG are still unknown [57].

A femtosecond pulsed infrared laser can be used to induce large index changes thanks to the high peak power [58]. The mechanism is different from the UV laser gratings since no color centers are involved. However, the absorption/ionization of a nonlinear multiphoton produces local physical damage, leading to increased density or the generation of physical defects. The properties of these gratings are similar to Type II induced by nanosecond UV lasers. This grating has better spectral performance. 

The advantage of this FBG is that it can be inscribed on many types of waveguides, not only silica-based UV-sensitive fibers. Therefore other materials can be used, e.g., sapphire, allowing the production of sensors for highly specialized tasks such as high-temperature sensing above 1750 °C on sapphire fibers [52].

#### 2.1.3. Long Period Gratings

Long period gratings are similar to FBG but with a period typically in the range of 100 μm–1 mm, many times larger than the typical wavelength propagating inside the fiber. LPG promotes the coupling of the light coming from the core to the propagating cladding modes [59]. Cladding modes have high attenuation; therefore, the spectrum presents an attenuation band for a specific wavelength related to the periodicity of the LPG. If the environment changes the period or refractive index of the section containing the LPG (typically around 30 mm), these attenuation bands will shift, similar to FBG. LPG sensors have been used for many applications in physical, chemical, and biological sensing [60,61,62,63,64]. The sensitivity for each measurand depends on the composition of the fiber and the order of the cladding mode coupled; therefore, it is possible to have a single sensor that senses multiple parameters.

There are many fabrication techniques for long period gratings to produce the periodic modulation of optical properties by modifying the core’s refractive index or by physical deformation of the fiber. These techniques are shown in Table 2, and the most common is the UV irradiation of germanosilicate fibers.

LPGs attracted the attention of researchers for their use in radiation environments. The first study of their behavior under γ irradiation was by Vasiliev et al. [76] in 1998. A complete review was conducted by Esposito et al. [77], showing the growing interest in these sensors while, at the same time, underlining the need for further studies on the influence of dose rate and irradiation temperature on their performance.

### 2.2. Distributed Optical Fiber Sensors

Distributed optical fiber sensors (DOFS) are meant as an improvement in spatial resolution attainable with quasi-distributed system (or multiplexed system) point sensors such as FBGs. The first DOFS system was based on the optical time domain reflectometer and allowed the spatial measurement of any environmental parameter that influenced the fiber’s attenuation with a spatial resolution ranging from 0.1 m to 10 m [78]. DOFS are based on a scattering phenomenon that occurs to light during the propagation inside the fiber optics. Currently, based on the used mechanism, three main technologies are available in DOFS: Rayleigh, Brillouin, and Raman. Other technologies exist, such as the optical carrier-based microwave interferometer, but they are infrequent in most of applications [79].

**Figure 9:**
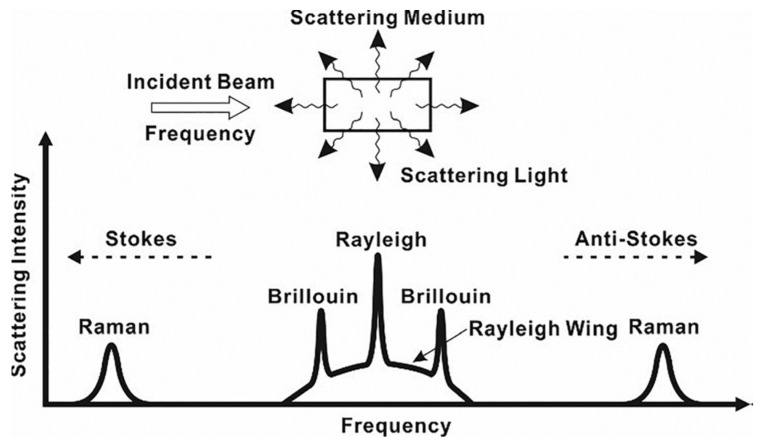
Light scattering spectrum with Rayleigh, Brillouin, and Raman scattering [79].

Scattering is a random statistical phenomenon that occurs in all angular directions, and in the case of elastic scattering, the frequency of the scattered light is conserved with respect to the input one (Rayleigh). Additionally, if the frequency of light or its energy changes during the scattering process by absorbing or giving energy to the fiber material, then the scattering is called inelastic (Raman and Brillouin). DOFS can thus be classified by what happens to the frequency of the incident light and the occurring scattering mechanism, as shown in Figure 9.

In Rayleigh-based DOFS, a propagating pulse of light in the fiber can maintain its central wavelength, while perturbations along the propagation broaden its optical spectrum. Such elastic scattering originates from material density fluctuations, dopant concentration, or fiber perturbation (strain or temperature) reaching the fiber’s core. Due to its isotropic nature, not all the Rayleigh scattered light goes in the same direction: part follows the initial propagation direction, and part is backscattered (against the propagation direction). These sensors use the backscattered light as in optical time-domain reflectometry (OTDR). When an external perturbation changes the density or dopant concentration locally, its intensity can be measured by the difference signature of the backscattered light before and after [79].

There are two approaches that use this phenomenon:

- Optical time-domain reflectometry is the simplest and measures the intensity of the backscattered light. Each measurand is dependent on the intensity, and the time of arrival of the sensed light is correlated to the distance propagated along the fiber.
- Optical frequency-domain reflectometry (OFDR) is more complex and is based on the analysis of the measurement of light over two polarization states obtained by the reference light. The position where the variation is happening is related via a complex elaboration of the optical frequency signal.

The advantage of OFDR is that it has greater spatial resolution along the fiber than OTDR, which is at the meter level. In addition, OFDR also has a large dynamic range at the expense of a more complicate and expensive interrogation system [80].

To overcome some of the shortcomings of these two techniques, a couple of less-used and still-developing approaches were used. Incoherent optical frequency-domain reflectometry is used to detect with a high spatial resolution over a long range. Indeed, a spatial resolution of 11.2 cm over 151 km was demonstrated [81]. Optical low-coherence reflectometry (OLCR) has been developed for the high spatial resolution measurement of small optical components with fine structures thanks to a higher than −95 dB reflection sensitivity [82]. It has micrometer-level resolution but a measurement range of less than a few meters.

Brillouin-based DOFS uses inelastic scattering given by energy transfer between photons and acoustic phonons. This interaction leads to a frequency shift of a quantity named Brillouin frequency that is proportional to the effective refractive index, the medium’s acoustic speed, and the incident light’s wavelength. The effective refractive index is the speed of light in a waveguide, such as an optical fiber, divided by the speed of light. This is the propagation speed of waveguide modes. Brillouin frequency varies between 9 and 13 GHz. The acoustic speed is related to the density of the medium; therefore, the change of density by temperature or strain is translated into a change of the Brillouin frequency. These sensors can be used to detect strain and temperature at the same time for tens of km with a typical resolution of 1–10 m.

Brillouin optical time-domain reflectometry (BOTDR) was first developed by Kurashima et al. [83] in 1993. This technique is single-ended and similar to Rayleigh’s OTDR but has few variations. Normally, direct detection is used as in Rayleigh DFOS, but there is also heterodyne detection, which employs a reference signal and allows for a longer sensing range and better performance, improving receiver sensitivity [84]. In the last years, there have been great improvements in BOTDR achieving great sensitivity for temperature and strain measurement. The maximum range achieved was 100 km, while the spatial resolution went down to 20 cm, and a minimum temperature shift of 0.37 °C and a deformation of 7.4 με were reported [79].

Another technique employed in DOFS is the Brillouin optical time-domain analysis (BOTDA), which is based on stimulated Brillouin scattering (SBS). In this configuration, the sensing pulse of light is injected on one end of the fiber, and at the other end, a counter-propagating CW laser is injected [85]. The detection technique is similar to standard OTDR systems, and in the same way, BOTDA achieved great improvements in the last years by reaching a longer sensing range of 150 km and a spatial resolution of 10 cm. Such improvements on the range and spatial resolution come at the expenses of the sensor sensitivity. Indeed, the maximum temperature sensitivity obtainable is 1.5 °C and the strain sensitivity of 20 με [79]. BOTDA obtained inferior ranges (up to 10 km) but a high spatial resolution of 2 cm, reaching a strain sensitivity of 70 με.

Another technique developed is Brillouin dynamic grating (BDG), which involves dynamically reconfigurable gratings. The BDG paired with PM fibers achieved sensitivities for temperature and a strain of 0.08 °C and 3 με, respectively [86]. By lowering the sensitivity, a spatial resolution of 20 cm was obtained [87].

Raman-based DOFS exploits an inelastic scattering phenomenon due to the interaction with optical phonons. As for Brillouin, the Raman shift is the difference in frequency between the pump photons and the scattered ones. Raman DOFSs are only sensitive to temperature and not strain and have been recently widely adopted for environmental monitoring [88]. Raman scattering has a small cross-section and thus tends to produce weak signals. Therefore, higher laser power must be used while avoiding stimulated Raman scattering. The most-used technique is Raman OTDR, and the highest spatial resolution achieved was of 1.2 cm with a sensing range of 2.8 m [89]. Commercially available Raman OTDR has a typical spatial resolution of about 1 m.

Raman OFDR is very similar to OTDR systems and can achieve 2 °C of temperature sensitivity, with a spatial resolution of 1 m on 16 km. The highest temperature accuracy reached was with an OTDR coupled with image processing that achieved 0.004 °C with a spatial resolution of 2 m [90].

### 2.3. Comparison of Point and Distributed Sensors

Point sensors with FBG gratings present an easy-to-use setup, a low cost, a small form factor, and a wide measurement range, but they are fragile by themselves, and typically, a package is required as protection. Typical FBG sensors have 1.21 pm/με which is relatively low, and in the last years, new advancements were made to increase it up to 31.4 pm/με, as Nawrot et al. [91] demonstrated in 2017. Zhang et al. [92] developed a Bragg strain sensor with a range of −1500–400 με for high-temperature applications. It is possible to multiplex a point sensor system to achieve quasi-distributed monitoring, but this increases the cost sensibly if large lengths are required to be monitored. The temperature application range for most of FBGs is <450 °C, but new femtosecond FBGs reached stability till 1000 °C, the highest among the OFS. The spatial resolution can be higher than other sensors with less than 1 mm resolution [93]. LPG sensors show higher mechanical strength compared to Bragg gratings but generally at a higher cost.

DOFS has the advantage of being already completely distributed, showing high sensitivity and fair precision. Each DOFS’s technology has a different resolution topping for Raman sensor at a resolution of 0.004 °C for temperature and 2 m of spatial resolution. Generally, distributed sensors have a spatial resolution of 1–10 m, with some reaching 100 m. The maximum temperature at which they are stable is normally much lower than for FBGs, and for regular fiber optics, it goes up to 600 °C. Only coherent Rayleigh can reach a spatial resolution in the order of mm. 

It is important to remark that, for all of these sensors, in harsh environment applications, it is absolutely necessary to combine at least two technologies to sense strain and temperature simultaneously along two different fibers. 

FBG sensors have been considered to substitute older electronic-based sensors in aerospace applications, but DOFS have found much more limited use in such applications. From a radiation response point of view, Bragg sensors have a great advantage: only the grating is the sensitive part, and the interrogation system, affected by radiation, can be installed far away. On the contrary, distributed fibers have much more delicate, sensitive, and expensive electronics, which is significantly more exposed in a radiation environment. In addition, for certain types of fibers (phosphosilicate), DOFS is strongly affected in the measurement along the fiber in the presence of radiation.

Overall, the main advantage of DOFS is the possibility of having a very long sensing fiber (up to tens of km), although for monitoring only a few critical points (up to hundreds), quasi-distributed systems are much simpler, cheaper, and more reliable.

## 3. Fibers and Optical Fiber Sensors in a Radiation Environment

**Figure 10:**
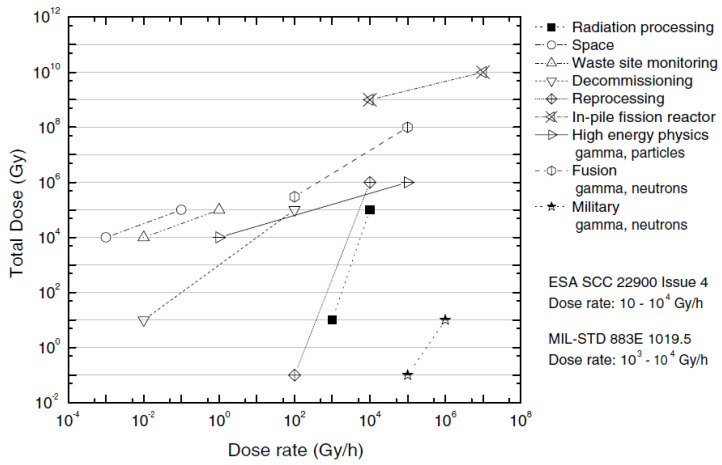
Overview of typical applications as a function of total dose and dose rate [98].

The advantages of the use of optical fibers for data transmission and sensing applications have sparked interest in their usage in a radiation environment. This fact has driven the studies of fiber optic radiation sensitivity in nuclear and space applications. Both applications are considered harsh environments, although they present different profiles in terms of radiation dose rates, atmosphere, and temperature conditions. The assessment of radiation damage of silica glass in harsh environments was initially made in the mid-1950s for amorphous silica in a nuclear reactor by Primak, Fuch, and Day [94]. However, only about forty years have passed since an extensive study on optical fiber behavior under irradiation was carried out [95,96,97]. In the context of fiber sensors for radiation environment applications, the radiation profiles present a multifaceted scenario. Figure 10 shows the typical radiation doses for different environments. In particular, we underline that space missions have low dose rates and modest total dose rates of radiation compared to fusion reactors. This fact places aerospace applications on a middle ground with respect to the requirements in terms of radiation tolerance between standard ground applications and extreme nuclear environments. 

Besides dose rates, different types of ionizing radiation must be considered when comparing space and nuclear environments. Protons, electrons, and heavy ions mainly compose space radiation, while the environment of nuclear applications has high doses of gamma and neutron radiation [99]. At the macroscopic scale, the main radiation effects that can impair the functionality of optical fibers and thus the related applications are radiation-induced attenuation (RIA), radiation-induced emission (RIE), and radiation-induced refractive index change (RIRIC).

**Figure 11:**
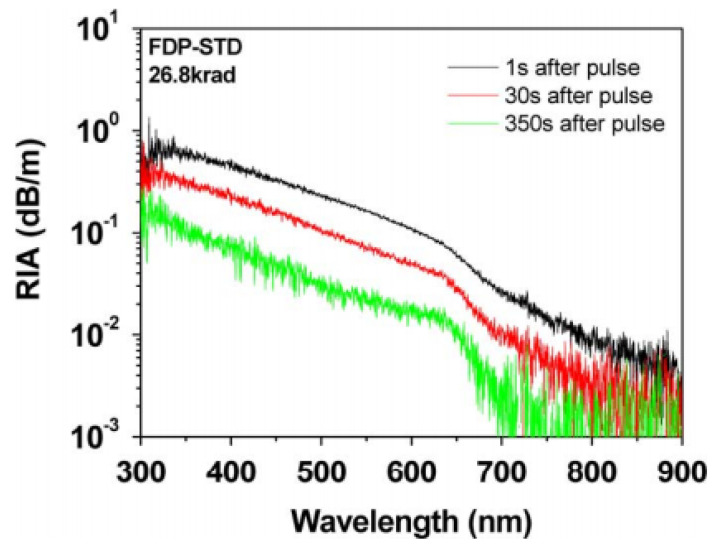
Spectral radiation dependance of the RIA at 3 different times of a solarization-resistant optical fiber from POLYMICRO, the FDP-STD fiber [100].

RIA appears during irradiation and consists of the fiber’s linear absorption increase due to the formation of new defects at the molecular level. This results in an increase in linear attenuation growing with the cumulative dose. Therefore, this process is time-dependent and wavelength-dependent and usually partially recovers after the radiation stops. An example of a typical evolution of this effect for different wavelengths and radiation times is shown in Figure 11 for different exposure times.

The RIA is normally calculated through this formula:

(3) $$\alpha_{\mathrm{RIA}}\;(\frac{\mathrm{dB}}{\mathrm{km}})=\frac{10}{L\;\left(\mathrm{km}\right)}\;\;\log\;\left(\frac{I}{I_{0}}\right),$$

with *I* and *I_0_* being, respectively, the intensity at a given time and before the pulsed exposure to radiation. RIA is commonly the main factor to consider in protecting fiber optics in a radiative environment because it can dramatically degrade the propagation distance. Indeed, an increase of losses from 0.2 dB km^−1^ to 2000 dB km^−1^ at 1550 nm has been reported for SMF28 fibers after only a few tens of nanoseconds of radiation [101]. It is interesting, anyway, to point out that, apart from being one of the significant limitations to the use of optical fibers in a harsh environment, RIA can also be used to obtain optical sensors for radiation sensing or dosimetry, as demonstrated by the DESY facility [102].

**Figure 12:**
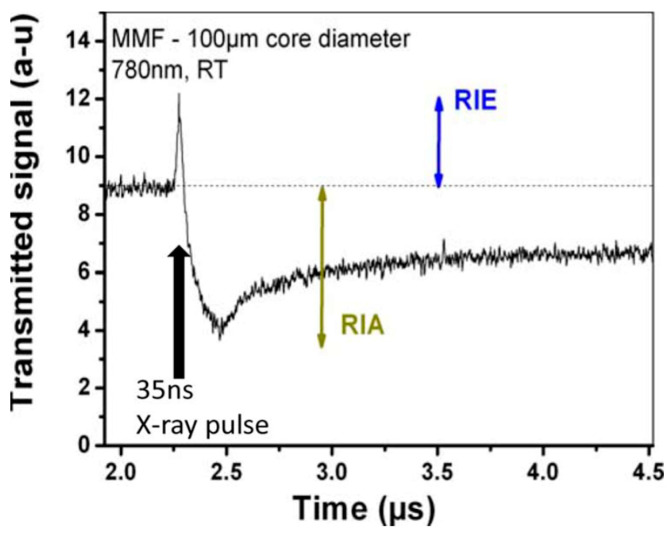
Radiation-induced emission and attenuation of a multimode fiber at 780 nm irradiated for 35 ns with 1 MeV X-rays [102].

Radiation-induced emission occurs when a sample under irradiation emits parasitic light that superposes on the propagated signal, as illustrated in Figure 12. RIE can originate from different mechanisms. Cerenkov light is observed only during the irradiation run for a high flux of sufficiently energetic particles. In addition to Cerenkov, luminescence signals from pre-existing defects and radiation-induced defects can be observed during irradiation too. RIE has an impact on fiber optic sensors since it affects the signal-to-noise ratio (SNR), increasing the noise levels, especially in the visible window and in an environment with a high dose rate (such as MegaJoule class lasers). As well as the RIA, the luminescence of RIE has been exploited to monitor the dose rate or the particle flux in different applications [101,103].

Two mechanisms cause radiation-induced refractive index change (RIRIC), density change of the glass matrix of the fiber according to the Lorentz–Lorenz [104] formula and the RIA described by the Kramers–Kronig relations, which define the relation between the refractive index and the absorption [105].

**Figure 13:**
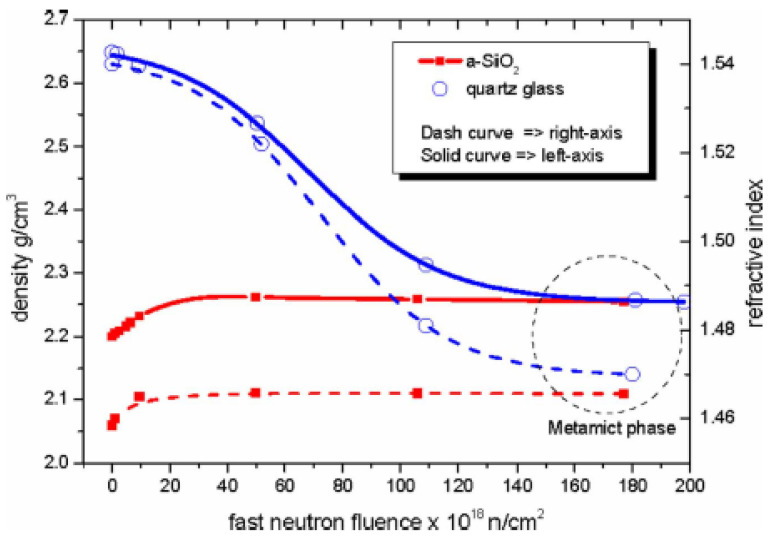
Density–refractive index relationship due to fast neutron radiation by [103] and adapted from [106].

The compaction or swelling of the glass leading to a change of the refractive index was first observed in bulk silica irradiated by fast neutrons by Primak [106]. The density change depends on the irradiated material’s phase. For instance, both amorphous and crystal silica phases change into the metamict phase [107] when irradiated by neutrons. Although amorphous silica has a 3% increase in density and RI, α-quartz decreases by more than 10%. The relationship between density changes and the refractive index caused by neutron irradiation in silica is shown in Figure 13.

RIRIC may also affects the optical waveguide structure leading to the appearance of new loss mechanisms [108]. At a microscopic level, it is well known that, when the radiation interacts with the glass matrix of the fiber, there are mainly two phenomena that create defects: knock-on damages, with the displacement of atoms, or ionization processes, depending on the energy of the incoming particle. For silica fibers, the matrix is mainly composed of Si and O atoms that need 18 eV and 10 eV to be knocked out of their original positions, respectively [109]. In ionization processes, instead, an electron–hole pair is formed by the electron jump from the valence band to the conduction band, leaving a hole behind. The formed pair can recombine radiatively, where at least a part of the energy is released as light emission or luminescence, or non-radiatively, in the form of phonons or secondary radiolytic processes that can generate point defects turning into new optical losses [110]. The radiation-induced color centers (or point defects) largely depend on the composition of the analyzed glass; therefore, reviews on this argument are continuously updated to summarize the evolution of the knowledge on this matter, and some examples can be found among these references [103,110,111,112,113].

The matrix of the fiber’s glass can already have defects such as dangling bonds or impurities, called precursor sites, and they disappear while the radiation-induced point defects increase through the same phenomenon, the trapping of radiolytic electrons and holes. Although every year there is an advancement in the knowledge of the kinetics of some defects, only a few defect types are fully known and currently can be studied. 

Nonetheless, a good amount of data for pure silica and G-doped or P-doped silica defects have been collected. Most of the precursor sites and radiation induced point defects absorb in the ultraviolet-visible band; therefore, the RIA will be higher for the UV and visible regions of the spectrum and lower for the near-IR. Unfortunately, the contribution of these defects depends also on their localization in the cross section, and their generation or bleaching are also affected by the internal stress of photobleaching. Confocal microscopy of luminescence (CML) and cathodoluminescence (CL) have been employed to study these mechanisms, showing promising results [114,115,116,117].

The RIA in IR is still under investigation because UV-visible absorption band tails do not account for all the optical losses. No absorption bands for silica fiber or Ge-Doped fiber are present in the IR; therefore, the defects causing this loss mechanism still need to be identified. For SM fibers in the IR domain, it is also important to evaluate the effect due to fiber-guiding properties on the RIA spectral dependence [103]. However, if the total dose continues to increase, the RIA will decrease instead due to the increased bleaching rate of its defects that outgrows their generation [103,118,119].

Most space missions in harsh environments have a long lifespan; therefore, experiments on RIA are made at accelerated dose rates to test for the target doses over the mission lifetimes. Griscom et al. [120] demonstrated that the dose rate could change the RIA for most fibers. Moreover, a faster dose rate increases the point defect generation but not the bleaching, resulting in a greater RIA.

**Figure 14:**
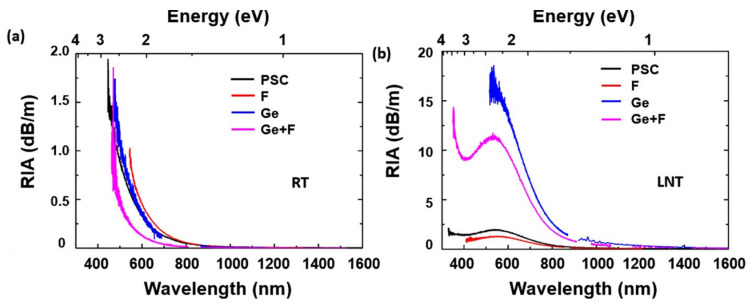
RIA under x-ray pulse after 1 s of different fibers: pure silica core (PSC OF), F-doped, Ge-doped, and Ge + F- doped fibers. (**a**) RIA at room temperature; (**b**) RIA at liquid nitrogen temperature [122].

The defect’s stability is affected by the temperature; therefore, the RIA changes considerably even with just a few degrees of temperature variation [121]. Figure 14 shows the RIA of different fibers at room temperature (RT) and at liquid nitrogen temperature (LNT). 

Most of the studies are made between −50 °C and 100 °C, roughly corresponding to the temperature ranges of the dosimetry and high-energy physics applications. However, space application needs a broader temperature range between −200 °C and 260 °C [123], and nuclear application needs even higher temperatures reaching 800 °C [124]. Only a few works have investigated such high temperatures while showing this study’s importance in understanding fiber behavior in different radiation environments. For example, RIA is divided by a factor of 100 at 400 °C for high-purity UV grade silica fibers [125,126] compared to room temperature, while in the cryogenic regime, the RIA was found to increase at temperatures as low as 77 K [127]. Temperature influence was also studied for pure-silica bulk glass irradiated by very fast neutrons [128,129] and for a variety of point defects: silica point defects [110], phosphorus defects [130], and germanium defects [131]. For aerospace conditions, only temperature response [132] or radiation response were independently investigated, and a comprehensive study comprising both variables still needs to be addressed.

Another phenomenon that can change the fiber behavior under irradiation is the power level of injected signal that, for high values, can cause photobleaching as reported by Henschel et al. [133]. Their work demonstrates how photobleaching is less important in more recently produced fibers and nearly negligible for Ge-doped telecommunication fibers.

Different experiments have been carried out on commercial SM and MM fibers in the near-IR [134]. Radiation tests were also conducted on prototype MM in the visible [135,136,137,138], while fluorine-doped fibers were tested at 1542 nm [139]. In the near-IR, aluminosilicate fibers were used as dosimeters [140] and were proposed as radiation-tolerant fibers. Studies on single mode emitters, fibers, and photodiodes with gamma radiations up to the MGy level were conducted by Berghmans et al. [141], obtaining low RIA even at 3 MGy of total dose with a dose rate around 3 kGy/h. For higher dose rates, such as 25 kGy/h, and higher temperatures, this is no longer true, with losses from 15 dB to 20 dB over 100 m long fibers. From a practical standpoint, standard optical connectors showed degradation, but fusion splices demonstrated far better resistance with an insertion loss of around 0.5 dB with 20 MGy of total irradiation, constituting acceptable losses for a small number of splices.

**Figure 15:**
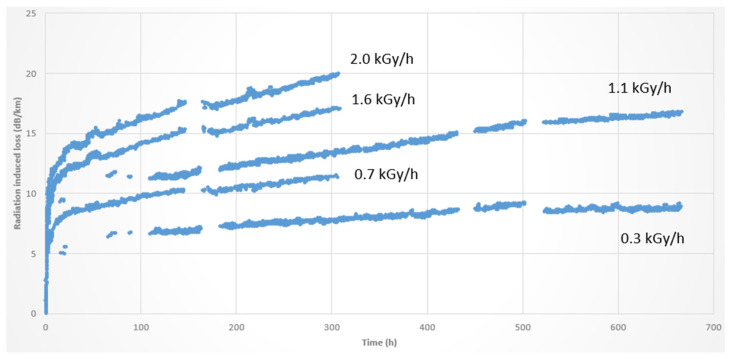
Radiation-induced loss at 850 nm in Spectran TCG MM fiber at different dose rates adapted from [142] fitted according to the power–law model of M. Van Uffelen et al. [143].

To illustrate the radiation-induced loss of a commercial-off-the-shelf (COTS) 100 μm core fiber, we report in Figure 15 its RIA over time for different dose rates (0.3–2 kGy/h) for a total dose of 1 MGy. 

Other measurements were made on COTS optical fiber, vertical cavity surface emitting laser diodes, LEDs, multimode photodiodes, and WDM components [142]. The optical losses originating from radiation from the different optoelectronic components, including fibers, can be considered in the system power budget and its performance over time.

**Figure 16:**
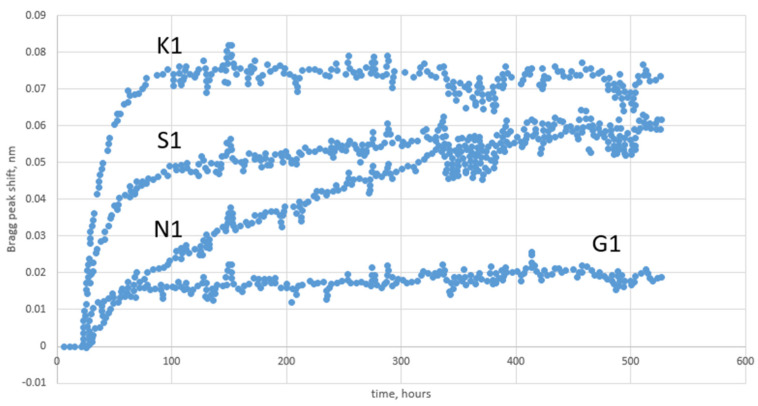
Shift of the Bragg peak under gamma radiation for different FBGs, adapted from [142].

Focusing the attention on optical fiber sensors, we must first underline one of the key advantages of using these sensors in harsh environments: the possibility of having only the sensing part (i.e., the fiber) exposed to radiation while the interrogation unit could be located far away in a more protected environment. This facilitates the study of the radiation tolerance of a sensor system, limiting the investigation to the optical fiber and avoiding studies on all the electronics. Concerning FBG sensors, radiation influences their responses in two ways. RIA degrades the grating performance by decreasing the SNR, lowering the peak until it appears undetectable [76]. In addition, RIRIC affects the Bragg peak, causing a decrease of peak reflectivity and a wavelength shift as illustrated in Figure 16 [144]. The main parameters influencing the radiation response of FBGs are fiber parameters, irradiation conditions, FBG types, and writing conditions [15].

**Figure 17:**
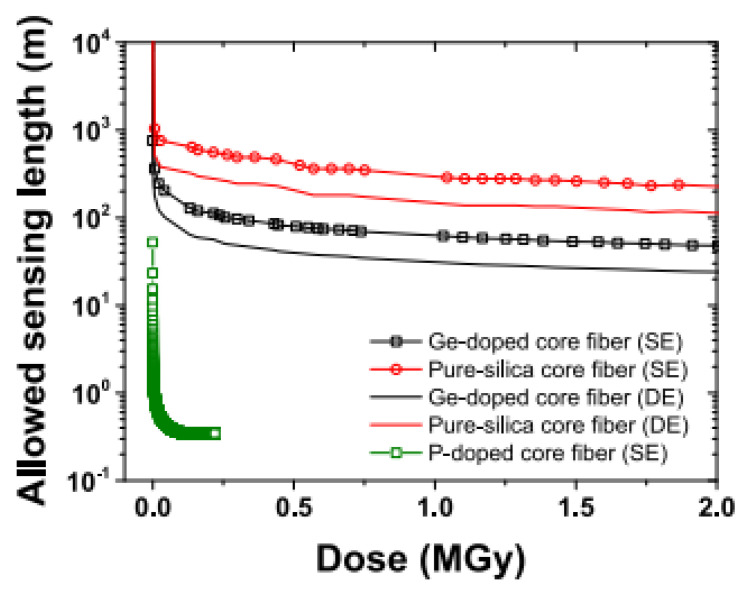
Sensing length decrease at increasing radiation doses for different compositions of optical fibers [15].

Under irradiation, distributed sensors suffer from a substantial reduction of the available sensing length caused by RIA. The entity of such reduction depends on the harsh environment, the fiber type, and its profile of use. In Figure 17, we report the decrease of sensing length for different kinds of optical fibers under irradiation for different doses.

In addition to this effect, it has been observed that radiation can change the structure of the silica glass, especially in case of high fluences of neutrons, inducing an error in the evaluation of sensor measurand (temperature, applied strain, etc.). For distributed sensors, Rizzolo et al. [145] demonstrated the success of in situ temperature measurements with a Germane-silicate commercial SMF28 fiber up to 1 MGy total dose of 10 keV X-rays, which did not alter the physical phenomenon of scattering. Studies on optical frequency domain reflectometry with a total dose of 10 MGy showed that the scattering properties are not affected, and their sensing mechanism works as in pristine conditions (without radiations). The RIA for radiation-resistant fibers (F-doped and pure silica core fibers) stays under 55 dB/km, which is acceptable for most of the applications of these classes of sensors. However, the overall sensing lengths for DOFS are generally significantly reduced for radiation environments. This could explain the preferred adoption of FBG/LPG sensors in this type of application.

## 4. Radiation Hardening of Optical Fibers for Aerospace Applications

To prolong the operational life of optical fibers and optical fiber sensors in a radiative environment, the strategy of choice is to “harden” them against radiations. For that, the two most commonly used approaches are hardening by component, where there is an improvement on the material by employing a new class of glass, and hardening by pretreatment, where the fiber core is loaded with a reactive gas that stabilizes against radiation. In this section, we give an overview of these two hardening strategies for completeness, while, for a more detailed treatment, the reader is encouraged to consult the reported bibliography.

### 4.1. Radiation Hardening by Component

To achieve the radiation hardening of fibers, a custom tuning of the composition must be implemented, in particular, fixing the target operational spectral domain and the environmental conditions. In fact, no optimal composition exists that allows the design of an optical fiber with a reduced radiation sensitivity for all harsh environments at all wavelengths. For instance, fiber’s composition with low RIA for infrared applications under γ-rays can have a poor response in other environments.

In this context, most of the research has been carried out for radiation environments up to a few MGy and for operating spectra from the visible to the IR. As examples, we here recall that fibers for plasma diagnostics in the ITER facility and fibers for the data links at the Large Hadron Collider have been thoroughly studied, driving the choice for these applications towards pure silica core (PSC) and fluorine-doped fibers [146,147,148]. To improve the RIA of PSC, the first step is to reduce impurities. Dry silica has a low impurity level but a higher amount of chlorine, which lowers the transmission in the UV. In comparison, wet silica has a higher amount of hydroxyl and a low chlorine content, which is better for ITER requirements. With chlorine and hydroxyl defects missing, the fiber obtained is worse due to the creation of new defects that are unstable even at room temperature, known as self-trapped holes (STH) that produce absorption bands in the visible range. 

For space applications, it is important to study doped core fibers used for several applications, e.g., Er-Yb doped fibers for amplifiers in space applications that use the co-doping with cerium [149] and Ge [150] to decrease the RIA by a factor of almost 25%.

### 4.2. Radiation Hardening by Pre-Treatment

A very efficient approach to the radiation hardening of fibers is the pre-treatment of the fiber core with a gas that reacts with the radiation-induced point defects and passivates them, producing more stable bonds that do not significantly affect the optical properties. For the PSC and F-doped fiber used in nuclear applications, the presence of hydrogen was reported to increase the performances in the visible spectrum by the passivation of the non-bridging oxygen hole center [148,151,152]. 

Similar to the hardening by component approach, drawbacks are also present in the gas passivation process. Indeed, hydrogen reacts with the oxygen of the Si-O matrix to create hydroxyl groups, whose presence increases the IR’s absorption and can combine to create new defects absorbing in the UV. The use of deuterium was also explored, giving some small benefits over H_2_ for signal propagation in some applications [153]. The main problem with hydrogen and deuterium is that, at room temperature, the gas diffuses out of the fiber in a few tens of hours, which makes it unable to be used for long missions in space environments. Oxygen has also been proposed as a solution because of its lower mobility. Indeed, oxygen can be added at high temperatures and pressure or as an excess dopant during the fabrication of the fiber, giving better RIA performances in the IR while negatively affecting the UV region [154,155,156].

Another pre-treatment used is pre-irradiation, which transforms in optically active point defects the precursor sites and then bleaches the defects. In this case, the approach exploits the fact that having fewer precursor sites gives a better response to irradiation, as demonstrated by Griscom [157,158].

### 4.3. Other Strategies for Radiation Hardening of Fiber

**Figure 18:**
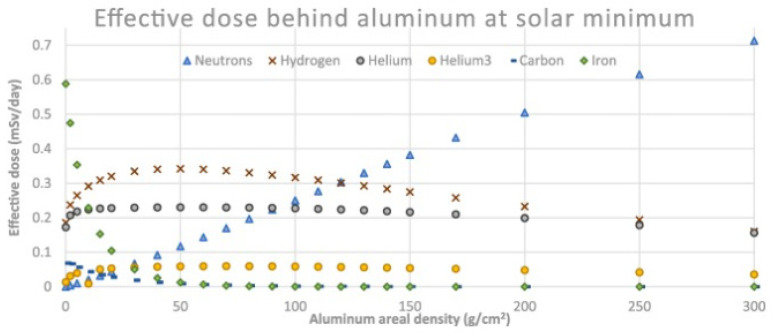
The effective dose of different ions at the solar minimum for various depths of aluminum shielding [161].

**Figure 19:**
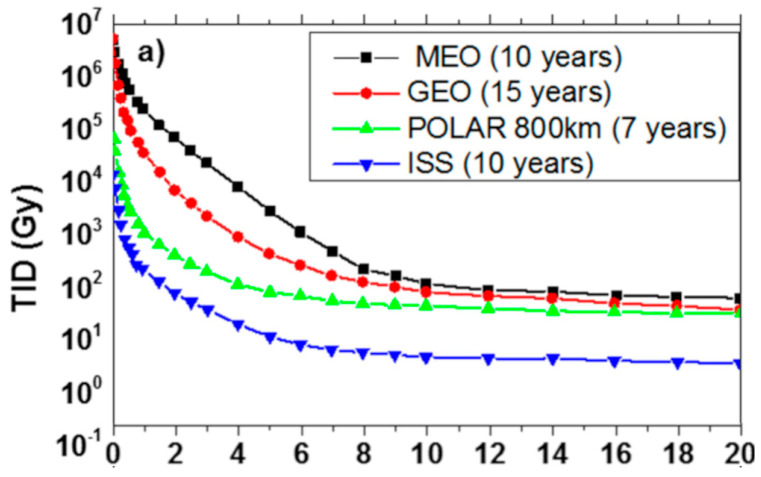
The total ionizing dose of an aluminum solid sphere with different thicknesses for various space mission [15].

Operating conditions greatly impact the radiation response of the fiber and might be easier to adjust than changing the fiber composition or using a radiation pre-treatment. In particular, the main parameter to be tuned is the operating optical signal region. Higher wavelengths in the IR spectrum are more desirable since fewer point defects produce absorption in that region [159]. When irradiated by steady-state γ-rays, the best performing wavelength for sensing is around 1–1.2 μm [160]. Aside from hardening the optical fibers, a classical shielding approach can also be used. Aluminum is a commonly used shielding material, and in Figure 18, we report the effective dose of different particles with respect to various amounts of aluminum in the shielding layer. Figure 19 reports the total ionizing dose calculated for a solid sphere of aluminum for space missions with different duration.

**Figure 20:**
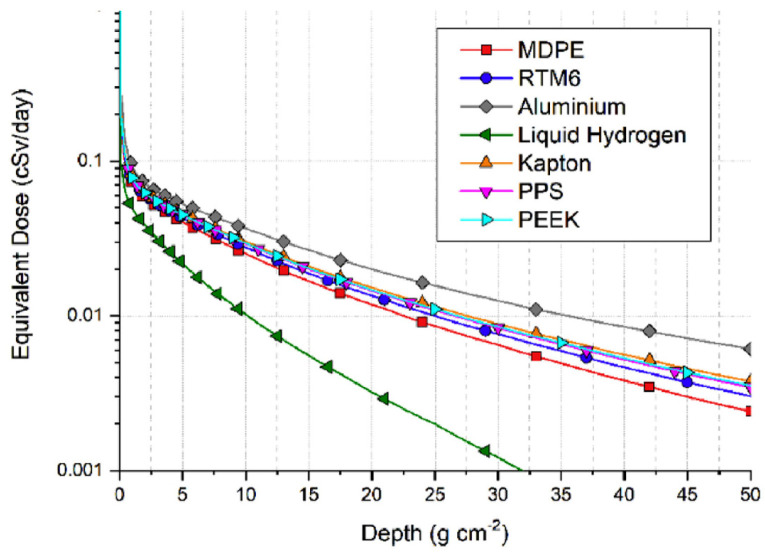
The equivalent dose of galactic cosmic rays for different materials in the LEO environment with an inclination of 51.6° recorded during 2015 [163].

Although aluminum is a commonly used material for shielding in space environment due to its characteristics, e.g., lightweight, and well-integrated with space constraints, its shielding performance is poor compared to other materials, as shown in Figure 20. Indeed, other materials such as some polymers (PPS, Kapton, PEEK) and even water can have much better shielding properties [162].

Shielding is a common solution for radiation protection for electronic devices or the astronaut’s safety, but it directly translates into adding materials that eventually increase the overall weight. For this reason, improving the radiation resistance of optical fiber sensors is still crucial, and a joint approach combining fiber composition and shielding could be an enabler toward the widespread use of fiber sensors in the aerospace industry.

## 5. Advantages of Fiber-Based Sensors

Optical fibers are suited for many aerospace applications thanks to their high bandwidth, multiplexing capability, and low attenuation level [46]. Thanks to their electromagnetic interference immunity, many aircrafts, such as the Eurofighter “Typhoon” and the Boeing 777, started to include optical fibers for on-board data communications since the 1990s. In the last twenty years, optical fibers have been considered not only for communication networks but also as sensors for different applications, particularly for the structural health monitoring of aircraft. Indeed, one of the most stringent constraints in aerospace applications is weight, and the lightweight optical fibers perfectly suit the requirement for lightweight devices [164,165].

The trend of the adoption of lighter construction materials such as fiber composites in aircraft manufacturing and the possibility of implementing prognostic functionalities on components implies that structural health monitoring is vital to improve safety and reliability while minimizing maintenance costs and manpower. Thanks to their small footprint, optical fiber sensors can be easily embedded in different materials, e.g., graphite/epoxy composites, as demonstrated for spaceships [166,167]. Electrical devices in space have two reliability problems: sensitivity to electromagnetic interferences and connectors. Optical fibers address thanks to both their natural immunity to the electro-magnetic environment and the possibility of making “daisy-chained” systems of multiple sensors along a single fiber with only one connector. As a comparison, we recall that single-axis electrical strain gauges have three connectors, decreasing reliability and occupying more space. Optical fibers are also resistant to high temperatures, and the coating, usually a polymer, can be designed to withstand the typical temperatures of aerospace vehicles. A polymer category that is of interest for aerospace applications is polyimide, which has high mechanical resistance and can resist up to 300 °C. If higher temperature resistance is needed, a gold coating can be used on the fiber to achieve operational temperatures of 800 °C. While the electrical resistive strain gages are limited in sensitivity due to electrical noise, OFS do not have this constraint and do not require continuous interrogation [168], thus requiring low power per sensor. As an extreme example, we recall that Zhu and Chen investigated how heat treatment and polymer coatings can influence and improve stability up to 1000 °C [169]. Moreover, Rayleigh scattering-based DOFSs have been demonstrated to sense strain and temperature simultaneously, inside furnaces, with greater accuracy than thermocouples [170].

Concerning the resistance of fiber optics in the space environment, there are already many different applications. As an example, we recall here that, in 1992, the Small Explorers Program (SMEX) proved optical fibers as a viable option for telecommunication by being at the heart of the optical fiber communication system that was still functional after 16 years [171].

Besides communication and sensing, other fiber optic-based applications were implemented in space, such as the Light Detection And Ranging (LIDAR) system used on the Bepi Colombo and SWARM mission and the Raman laser spectrometer for the ExoMars rover in 2020. Many applications of OFS can be found in the aerospace sector, and the most significant examples are reported below.

### 5.1. Structural Health Monitoring

Structural Health Monitoring (SHM) aims at continuously monitoring structures for damage, improving reliability, safety, and maintenance costs while reducing human intervention. Indeed, not only is this useful for fault detection but also for predictive maintenance. Different studies revealed the positive impact of SHM [172,173] through fiber Bragg gratings point sensors or interferometers distributed sensors. Health diagnostic is especially interesting to implement in the design of new reusable launch vehicles and for the new composite materials employed in the aerospace industry where it is important to monitor stress factors. For example, the Thermal Protection System (TPS) is resistant to high temperatures and has high strength but has a brittle fracture mechanism, and a single fracture can lead to a catastrophic outcome, as in the Columbia shuttle tragedy.

Real-time sensing of multiple characteristics, such as mechanical stresses, temperature, and vibrations, is an upgrade compared to traditional nondestructive evaluation inspections. Besides the higher monitoring accuracy and immediate response to critical situations, it decreases the wearing of the structure caused by repeated disassemble and reassemble cycles.

**Figure 21:**
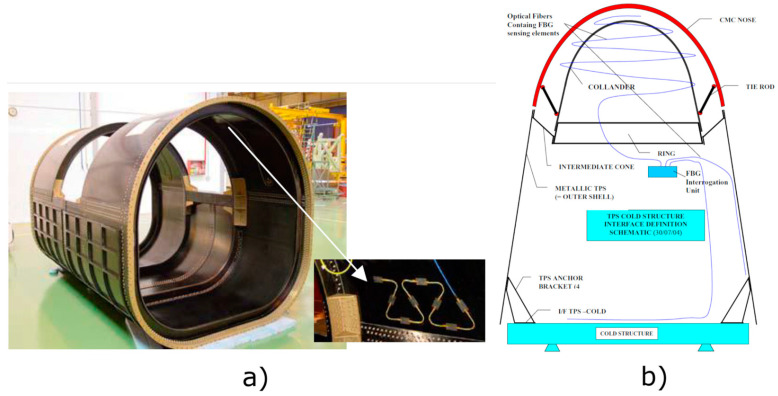
(**a**) Embedded fiber OFS on a demonstrator structure for an SHM (courtesy of EADS, CASA, and CONTRAVES SPACE) and (**b**) scheme of the SHM of the EXPERT vehicle [178].

SHM is a process that consist of multiple steps. First, periodic readings of the structure are taken with sensors using sampled dynamic response measurements. Then, damage-sensitive features are extracted to make a statistical analysis to determine the current state of health. Finally, a periodic update of aging and degradation effects on the structure is realized [174,175]. SHM is a key technology also for intelligent unmanned vehicles [176,177]. Different applications are currently being studied, such as strain sensing of solar sails. Coupled with actuators, they can monitor the deformations of the tripod typical of a telescope structure and counteract deformations. The analysis of deformations caused by strain and temperature of EXPERT atmospheric entry vehicle is also undergoing [178]. In Figure 21, we report two examples of OFS applications in structural health monitoring systems, showing their placement in critical parts of the aerospace structure.

Another successful example is the European FP7 PEASSS (Piezoelectric Assisted Smart Satellite Structure) project that created a 3U CubeSat with an FBG interrogator and 6 FBG sensors for strain and temperature sensing. It was demonstrated to work and resist without problems up to 4 MGy [179].

### 5.2. High-Temperature Sensing

Space vehicles go through a temperature range of −150 °C to 2000 °C, but available unpackaged commercial fibers used for temperature sensing can withstand only 300 °C [180]. New packaging designs have been developed in the last years to increase the maximum operating temperature of these sensors to 1000 °C, employing sapphire-based fibers with femtosecond written gratings [132,181].

Such a temperature sensor has the advantage of being distributed and can therefore improve the thermal management system, such as monitoring heat pipes as demonstrated by Kabashima et al. [182]. Another advantage is that multiple systems can be monitored with an exclusive channel without increasing the weight. The same fiber optic sensor system can have temperature and strain sensors without additional cost.

An example of a high-temperature sensing application is the PROBA II ESA mission, where the temperature goes from −40 °C to 400 °C and FBG sensors are used. 

### 5.3. Pressure Sensing

Pressure sensors are widely employed in aerospace, especially high-temperature ones, constituting a strongly growing market. Indeed, Alliedmarketresearch reported [183] a market size of USD 11.38 billion in 2019 and a projected USD 24.84 billion by 2027 with a significant increase of optical pressure sensors. 

Typically, pressure sensors nowadays are based on microelectromechanical systems due to their higher performance, lower power consumption, and compact footprint [184]. Optical MEMS pressure sensors have been studied since 1996 [185,186], but in recent decades, silicon fiber Fabry-Perot (FP) pressure sensors have attracted more interest, and they have been used successfully and with the advantage of being mass producible [187]. However, an important advantage of OFS sensors with respect to those is that they can be permanently integrated into the spacecraft and withstand higher radiation levels, thus making them promising for ground qualification and monitoring during the mission.

**Figure 22:**
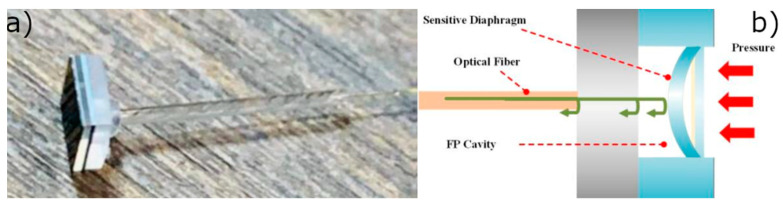
(**a**) Photo of pressure sensor for high temperature environment and (**b**) schematic design of its working principle [188].

Feng et al. recently reported a fiber-coupled Fabry-Perot sensor based on MEMS and CO_2_ laser fusion technology and analyzed it in the temperature range of 20–400 °C with a pressure range of 1 mPa and maximum nonlinearity of less than 1%. This sensor also has advantages in production thanks to its low cost and highly uniform response [188]. In Figure 22, we report the scheme of such a pressure sensor integrating the fiber optics and an FP cavity.

### 5.4. Vibration Sensing

Fiber optics vibration sensors can be employed for classical vibrometry and as indicators for particular failure modes of the system through the analysis of their specific spectral response change. In the last case, these sensors represent an excellent tool for prognostics, allowing the realization of predictive failure analysis, as shown by Quattrocchi et al. [189]. Indeed, characteristic vibrations can inform about the incoming failure, but they can also be an obstacle, for example, when an accurate pointing of the optical payload is needed. This is especially true for composite structures integrating PZT actuators that can actively dampen the vibrations. Such an approach is analogous to the strain sensors used to counteract deformations on telescopes and antennas.

### 5.5. Navigation Systems

Optical gyroscopes have substituted traditional mechanical ones because they are lighter, consume less power, have short reaction times, have a higher accuracy, and are mechanically robust [178,190]. They are based on the Sagnac effect in a fiber optics loop, which states that, in a closed ring path, counterpropagating beams allow the detection of the rotation with respect to an inertial reference. While commonly used for navigation on earth, their advantage has been recently demonstrated for satellites in the Sloshsat- FLEVO, which integrates a 3-axis FOG [191]. 

FOG uses different kinds of fibers, such as rare-Earth doped optical fibers (REDFs) rather than silica fibers. Coupled with an accelerometer, they can provide rotational speed measurements and can be used to give inertial positioning. FOGs are used at present in many state-of-the-art satellites, such as Altitude and Orbit Control System (AOCS) for planetary landing or for electric propulsion and deep space exploration. 

Recently, there have been many advancements in improving the effectiveness of FOG. To amplify the Sagnac effect, state-of-the-art FOG uses several kilometers of fiber per coil [192]. The main problem of such an approach is the increased vulnerability to radiation due to the longer fibers employed. The optical coil comprises several kilometers of fibers where the RIA effect quickly escalates. Moreover, the optical source uses an REDF (typically an erbium-doped fiber), which is more sensitive to radiation due to rare-earth doping. A radiation hardening strategy of FOGs is thus crucial, and it has already shown its effectiveness in many satellites such as Planck or Galileo [15].

### 5.6. Hydrogen Leak Detection and Chemical Sensing

Chemical sensing through OFS is useful in harsh environments and has been implemented for different chemical elements, although in space, the most compelling application is related to hydrogen detection. In fact, every leak can be catastrophic and it is very difficult to accurately pinpoint its location. OFSs are made of dielectric materials and thus produce no arc or spark, being intrinsically safe to be used in an environment with gaseous H_2_. 

In this case, chemical sensors can be based on FBG, LPG, interferometer, absorption intensity, and surface plasmon resonance (SPR). Bragg gratings cannot detect hydrogen, but if the fiber is properly coated with a material such as palladium or tungsten oxide [45,193], they can detect the elongation and bending of the reactive coating in the presence of hydrogen, and the concentration can be inferred by this change.

As reported by Zhang et al. [45], for chemical sensing, intensity based sensors are fragile and with low performance and, for this reason, are rarely used. FBGs are preferred over distributed sensing for their low cost, multiplexing capability, and small size, but they are fragile and can attain only moderate precision, while LPG has a higher cost, and the sensor can be mainly interrogated in transmission, but they are more robust and sensitive. 

All these sensors are affected by the same issues of standard optical fibers since the coatings applied for hydrogen leak detection or chemical sensing are typically made of heavy metals and they do not show significant refractive index changes in these coating upon typical radiation of the aerospace environment.

### 5.7. Radiation Sensors

OFSs, especially based on phosphorus-, aluminum-, or rare earth-doped fibers [194,195], can detect the presence and the dose of radiation if combined with a reflectometer. Indeed, the fiber degradation mechanisms in the presence of radiation are dose-dependent, and upon careful calibration, OFS can be used as a dosimeter. P-doped fibers for OFDR dosimetry can measure up to 100 kGy, with an accuracy of up to 20%, remotely and with a resolution of 15 cm under 10 kGy [196,197]. FBGs written in pure silica core fibers have also been used for remote dosimetry and temperature sensing. The principle is to measure the gamma radiation-induced blue shift of the Bragg wavelength. A dose of 100 kGy causes a shift of 20 pm [198]. Moreover, Yukihara et al. recently demonstrated their functionality in space application dosimetry [199].

There are two types of radiation sensors: intrinsic and extrinsic. The former uses optical absorption and the RIE phenomenon, while the latter uses scintillating materials. Research on intrinsic sensors is going on to check the stability and reproducibility of the irradiation-induced effects and for reusable sensors. At the same time, for the extrinsic sensors, the development is focused on improving the uniformity of the luminescence signals [200].

**Figure 23:**
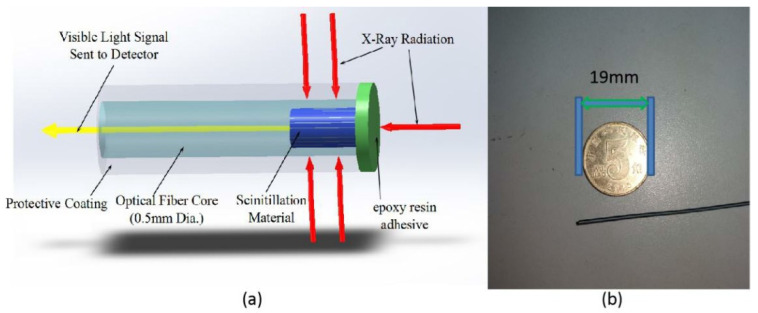
(**a**) Schematic representation of a scintillating-based fiber optic dosimeter and (**b**) the photograph with dimensions comparison [201].

An example of a novel fiber optic dosimeter based on an inorganic scintillating material integrated into a fiber is reported in Figure 23.

**Figure 24:**
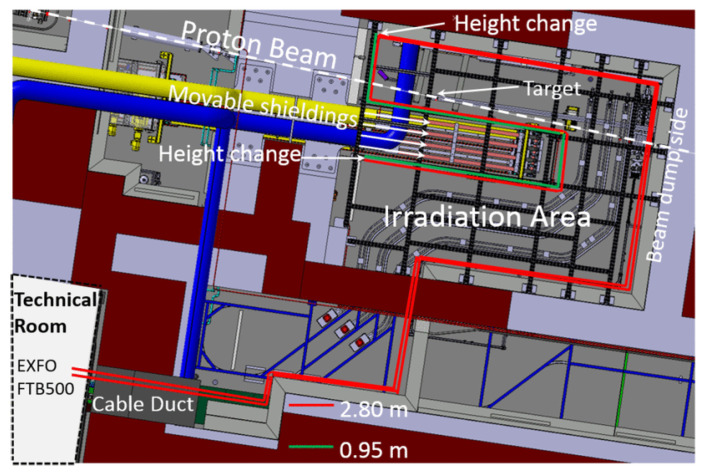
Distributed dosimeter in the CERN Proton Synchrotron Booster (PSB) [202].

For distributed dosimetry applications, OTDR systems are more suitable than OFDR because the detection of RIA occurring in the fiber is less complex [197]. An example of already employed DOF radiation sensing is found in the Proton Synchrotron Booster at CERN and is illustrated in Figure 24.

## 6. Conclusions

In this review, we reported on the recent applications of optical fiber sensors with their performance and radiation response. OFS operation principles and radiation damages were discussed, along with their best performances reported in the literature. At present, the radiation response of optical fibers is too complex and unknown to predict without experimental measurement the response to radiation for each kind of sensor. Nonetheless, OFSs have obtained promising results for the most common fibers in space missions. In addition, optimal results for several applications on earth pushed the case for their usage in future space missions and spacecraft. OFS will play a major role in aerospace applications, which will need accurate monitoring for unmanned vehicles and to increase the security of the manned ones. Despite a promising future, research needs to improve the knowledge about the radiation response of optical fiber composition and sensors. At the same time, there is a need for new packaging that can protect these sensors from space radiation sufficiently to increase their operational lifetime. In this direction, combining the work on fiber composition and the integration of shielding materials in sensor packaging can be a successful path toward the next generation of radiation-resistant aerospace fiber optics sensors.

## Figures and Tables

**Table 1:** Summary of principal space environment effects on space hardware [11,16,17].

Environmental Cause	Hazards	Effect
Structure impact	Micrometeoroids and debris	Structural damages and decompression
Surface erosion	Micrometeoroids, contamination, atomic oxygen, particle radiation, and UV radiation	Degradation of optical, electrical, and thermal properties and structural integrity
Total ionizing dose and dose rate	X-rays, γ-rays, protons, electrons	Degradation of microelectronics and optical fibers
Displacement damage dose	Heavy ions and neutrons	Degradation of optical components, solar cells, and some electronics
Temperature	Radiation, atmosphere drag, and space asset temperatures between −200 and 600 °C	Materials dilatation and degradation of mechanical, electrical, and optical components
Surface charging	Dense, cold plasma, hot plasma	Physical damage, power drain, and biasing of instrument reading
Internal charging	High energy electrons	Electrical discharges and biasing of instrument reading
Single event effect	Trapped protons and electrons, solar protons and neutrons	Electronic component damage, system shutdowns, noise on images, and data corruption
Other constraints	Vacuum, water, hydrogen, and other liquids and gases	Degradation of mechanical, electrical, and optical components

**Table 2:** Summary of principal fabrication techniques for long period gratings.

Production Techniques	Reference
Ultraviolet irradiation	[65]
Ion implantation	[66]
Irradiation by femtosecond IR laser	[67]
Irradiation by CO_2_ laser	[68]
Diffusion of dopants in the core	[69]
Relaxation of mechanical stress	[70]
Electrical discharges	[71]
Mechanical deformation	[72]
Tapering of the fiber	[73]
Core deformation	[74]
Clad deformation	[75]

## Glossary

AOCS	Altitude and Orbit Control System
BDG	Brillouin Dynamic Grating
BOTDA	Brillouin Optical Time Domain Analysis
BOTDR	Brillouin Optical Time Domain Reflectometry
CERN	Conseil Européen pour la Recherche nucléaire
CL	Cathodoluminescence
CML	Confocal Microscopy of Luminescence
COTS	Commercial Off The Shelf
CW	Continuous Wave
DOFS	Distributed Optical Fiber Sensor
EPR	Electron Paramagnetic Resonance
ESA	European Space Agency
FBG	Fiber Bragg Grating
FOG	Fiber Optic Gyroscope
FP	Fabry-Perot
Fs-IR	Femto second Infrared
GCR	Galactic Cosmic Rays
GEO	Geosynchronous Earth Orbit
GTO	Geosynchronous transfer orbit
IR	Infrared
ITER	International Thermonuclear Experimental Reactor
LED	Light Emitting Diode
LEO	Low Earth Orbit
LIDAR	Light Detection And Ranging
LNT	Liquid Nitrogen Temperature
LPG	Long Period Grating
MEMS	Micro Electromechanical System
MEO	Middle Earth Orbit
MM	Multi-Mode
MOF	Microstructured Optical Fiber
NASA	National Aeronautics and Space Administration
OFDR	Optical Frequency Domain Reflectometry
OFS	Optical fiber sensor
OLCR	Optical Low Coherence Reflectometry
OTDR	Optical Time Domain Reflectometry
PBGF	Photonic Bandgap Fiber
PbP	Point by Point
PhM	Phase Mask
PMF	Polarization Maintaining Fibers
PSCOF	Pure Silica Core Optical Fiber
PSB	Proton Synchrotron Booster
PZT	Lead Zirconate Titanate
REDF	Rare-Earth Doped Fiber
RI	Refractive Index
RIA	Radiation Induced Attenuation
RIE	Radiation Induced Emission
RIRIC	Radiation Induced Refractive Index
RT	Room Temperature
SBS	Stimulated Brillouin Scattering
SHM	Structural Health Monitoring
SM	Single-Mode
SMEX	Small Explorers Program
SNR	Signal to Noise Ratio
SPE	Solar Particle Event
SPR	Surface Plasmon Resonance
STH	Self-Trapped Holes
TID	Total Ionizing Dose
TIR	Total Internal Reflection
TPS	Thermal Protection System
UV	Ultraviolet
WDM	Wavelength Division Multiplexing
